# Lost and found: trends in litigation and compensation related to retained surgical foreign bodies

**DOI:** 10.3389/fmed.2025.1526271

**Published:** 2025-04-01

**Authors:** Simone Grassi, Martina Focardi, Francesco Santori, Marta Guerini, Elisa Ferri, Giulia Ferretti, Ilenia Bianchi, Filomena Autieri, Vilma Pinchi

**Affiliations:** ^1^Laboratory on the Management of Healthcare Incidents (LOGOS), Department of Health Sciences, University of Florence, Florence, Italy; ^2^Section of Forensic Medical Sciences, Department of Health Sciences, University of Florence, Florence, Italy; ^3^Accreditation, Quality and Risk Management Unit, Careggi University Hospital, Florence, Italy

**Keywords:** retained surgical foreign bodies, sentinel event, legal medicine, medical malpractice, never event, gossypiboma

## Abstract

**Introduction:**

Retained surgical foreign bodies are supplies and devices unintentionally left at the surgical site. They are generally considered never events, albeit even full compliance with procedures can only minimize the risk of their occurrence. As never events, affected patients often allege gross negligence, and hospitals are often forced to compensate for the damages. Despite the fact that the physical consequences of the retention are usually mild and temporary, and thus the compensation paid may be hypothesized to be correspondently low, clear data on the medico-legal outcomes of these claims—both extrajudicial and judicial—and the average compensation have not yet been described.

**Materials and methods:**

This paper presents a retrospective study on the related claims received between 1 January 2010 and 30 May 2024 by a large university hospital in Florence (Italy). The study aimed to deduce their incidence and mean costs, as well as the risk of medical malpractice claims leading to criminal complaints.

**Results:**

We identified 27 eligible cases, with a mean compensation of €20,695.49. During the same period, the claims unrelated to retained foreign bodies, used as controls, had a mean compensation of €67,542.26. When considering only non-fatal events, criminal lawsuits were present in 12% of the cases compared to 6% in the control group, which fell within the same compensation range. The majority of the cases (63%) were directly managed by the hospital, although this was a lower percentage compared to the control cases (76%).

**Discussion:**

In conclusion, even if the economic dimension of claims related to retained surgical foreign bodies is relatively contained, they are associated with a 2-fold risk of criminal lawsuits for doctors. In addition, patients are less confident about out-of-court settlements provided directly by hospital committees compared to judicial court trials. This indicates that patients perceive a retained surgical foreign body (RSFB) as a never event, which requires less justification compared to other wrongful medical care incidents. This perception is likely driven more by a breach of trust in doctors and hospitals than by the severity of consequences, which are typically mild or limited to temporary impairment.

## 1 Introduction

Retained surgical foreign bodies (RSFBs) are supplies and devices that are unintentionally left in the surgical site. These can include items that are usually counted after a procedure (e.g., sponges, towels, and sharps) and fragments of instruments and devices (e.g., a broken tip of a needle or catheter and a piece of a surgical device) (1). They are usually considered “never events,” i.e., events that are considered generally preventable. However, their incidence is still relatively high (up to 1.0 per 700 procedures) (2). Moreover, the incidence of RSFBs is thought to be underreported due to often delayed clinical signs and because not all RSFBs actually qualify as sentinel events—e.g., unretrieved device fragments are usually not reported (3). To date, abdominal surgery and gynecology have emerged as the most affected medical specialties, and surgical packs/sponges (in particular, surgical and vaginal sponges) (4, 5), followed by drain tubes and vascular devices (6, 7), are the most frequently RSFBs (8, 9).

Some clinical determinants of RSFBs are known, such as complex, emergency, unplanned, or prolonged surgical procedures, high body mass index, and the use of large sets of surgical instruments (10). However, the risk of RSFBs mainly depends on organizational factors, such as absent or unobserved preventive procedures, no or incorrect surgical count, or cognitive and human factors such as team communication and situational awareness (11). Although instrument counting at the beginning, during, and end of the procedure is considered the best preventive measure, most RSFBs occur after procedures with a correct count (12). Other proposed corrective interventions include taking radiographs of the surgical field immediately before or after fascial closure in the case of incorrect counting, using barcodes/radio-frequency identification tags for soft materials, and using magnetic retrieval devices and sharp detectors for metallic items (13–15). Since RSFBs are considered never events, medical malpractice is often claimed as gross negligence. Nevertheless, the reported compensations related to these cases vary widely, ranging from 37,041–2,350,000 to 150,000–5,000,000 US dollars per case (1).

All the medico-legal issues associated with RSFBs and the expenditure on related compensation to patients are currently underreported and under-discussed. Therefore, this retrospective study aimed to analyze the incidence and characteristics of medico-legal claims related to RSFBs that occurred at Careggi University Hospital, a public tertiary hospital in Florence, Italy. The study sought to compare these specific compensation claims with controls—i.e., medical malpractice claims unrelated to RSFBs. The primary endpoint was to identify trends in the incidence and costs of these specific claims, while the secondary endpoint was to evaluate if RSFBs correlate with a higher risk of healthcare personnel facing criminal court proceedings.

## 2 Materials and methods

Following approval from the Ethics Committee (code: “n.24059_oss, date 19/03/2023”), a retrospective analysis of medical malpractice claims related to RSFBs at Careggi University Hospital (Florence, Italy) was conducted for the period from 1 January 2010 to 30 May 2024 (the years of the claims).

The inclusion criteria were as follows:

- The retained foreign body was confirmed as an RSFB.
- An RSFB was alleged by a patient who filed a medical malpractice claim against the hospital for possible compensation.

The exclusion criteria were as follows:

- RSFB occurred, but the patient did not request compensation from the hospital.
- RSFB occurred and was reported according to incident reporting procedures, but the patient did not file a claim for compensation.

As for the controls, we considered the medical malpractice claims unrelated to RSFBs received by the hospital during the same period.

While analyzing the legal and clinical documentation available for each claim, different variables were considered, including age, sex, type of procedure, and type of foreign objects/medical devices. Moreover, the intervention of the risk management service of the hospital, the medico-legal evaluation, case assessment, and out-of-court settlements Legal Medicine analyzed. The examined hospital fully retains the medico-legal risk emerging from litigation with patients, without any insurance coverage or external assistance in handling complaints and claims. These are managed by an in-hospital Medical Malpractice Claims Management Committee (MCMC), composed of medical experts in legal medicine, loss adjusters, lawyers, and the hospital’s risk manager (16, 17). The MCMC can operate in three different scenarios, corresponding to the three options available in Italy for a patient to claim compensation for damages caused by a hospital:

- C1: The patient files a claim alleging hospital liability and related damage. The claim triggers the MCMC intervention, a negotiation with the claimant that can typically end either with an out-of-court settlement or with a rejection.
- C2: The patient directly opts for mediation by turning to authorized mediation bodies. A mediator tries to facilitate the composition of the litigation.
- C3: The patient directly opts for civil court proceedings. If no mediation was previously undertaken as described in C2, National Law n. 24/2017 allows the patient to file a special civil action called preventive technical inquiry. According to this special civil court proceeding, the judge appoints one or more medical experts who initially act as conciliator(s). In the event of failed conciliation, the experts write a report as court-appointed experts, answering the judge’s questions regarding medical and hospital liability and related damages.

All these variables were analyzed for both RSFB-related claims and control cases, i.e., claims unrelated to RSFBs.

To compare the mean compensation, we excluded fatal cases from both groups because, in Italy, compensation for wrongful death is highly variable, depending on multiple and heterogeneous factors, such as family members or heirs entitled to receive compensation for the death of the patient.

Regarding the secondary endpoint, a comparison was made of the incidence of criminal complaints in non-fatal cases for both the RSFB-related sample and the control sample (RSFB-unrelated cases). Both cases and controls were selected if they fell within the same economic range of compensation. Indeed, by limiting the analysis to relatively low (and comparable) compensations, we aimed to exclude the possibility that the decision to file a criminal complaint was mainly driven by economic factors or by severely invalidating health consequences. Since fatal cases were excluded from both samples (RSFB-related and RSFB-unrelated), the study also excluded cases of possible culpable homicides, for which reporting to the public prosecutor is mandatory and criminal proceedings start without any action from the patient’s relatives. The non-fatal bodily injuries considered here may integrate the crime of culpable personal lesions, which in Italy can only be prosecuted when the plaintiffs file a criminal lawsuit, thereby initiating the criminal proceeding. The rate of criminal lawsuits for personal lesions against healthcare personnel, as well as the preference to directly turn to civil court for claiming compensation for an RSFB, can be considered risk indicators of intense adversarial litigation between patients and healthcare personnel and/or hospitals.

## 3 Results

**Figure 1:**
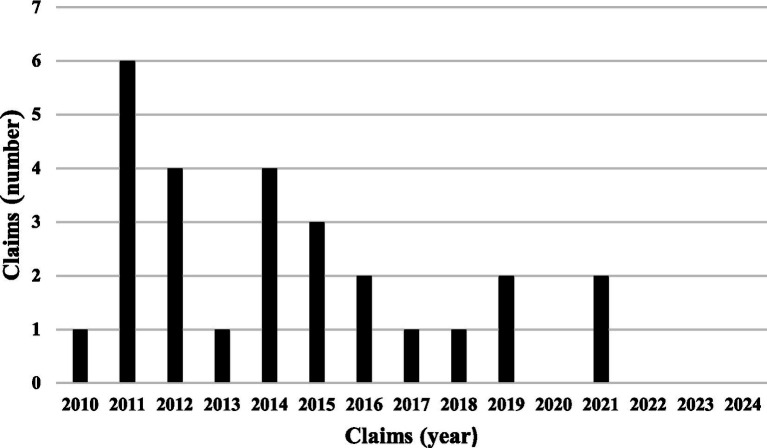
Litigation distribution.

We identified 27 eligible cases, half of which were reported to risk management, while a single RSFB case was excluded because it was reported but not claimed. The paid compensation ranged from €0 to €102,481.41 (Figure 1). Only one of the 27 cases was fatal.

During the same period, we identified 1,160 eligible controls, of which 646 were non-fatal cases with paid compensations not exceeding €102,481.41—consistent with the compensation amounts for such cases.

In the RSFB group, age ranged from 31 to 77 years (mean age: 50.8 years; median age: 49.5 years), and the predominant sex was male (17 male vs. 10 female individuals). In the control group, age ranged between 0 and 96 years (mean age: 52 years; median age: 53 years), and the predominant sex was female (629 female vs. 531 male individuals).

Regarding the cases, approximately a fourth of them (seven cases) occurred during orthopedic procedures, followed by emergency surgery (five cases), neurosurgery, in particular, spine surgery (four cases), gynecology-obstetrics (three cases), abdominal surgery (two cases), and oncological procedures (two cases). Urological, maxillofacial, cardiothoracic, and vascular surgeries share the same prevalence (one case each). Most of the claims (cases) were related to elective surgery (19 cases).

Approximately two-thirds (63.0%) of the retained foreign bodies were sponges, while in 22.0% of the cases, the object was a broken part of a surgical instrument (Table 1).

**Table 1:** Typology of the retained foreign bodies.

	Age	Sex	Year of the incident	Year of the claim	Retained foreign body
Case 1	67	M	2021	2021	Sponge
Case 2	39	M	2019	2021	Broken endovascular catheter
Case 3	51	F	2018	2019	Sponge
Case 4	68	F	2018	2019	Sponge
Case 5	70	M	2016	2018	Broken spinal catheter
Case 6	55	M	2016	2017	Surgical forceps
Case 7	59	F	2009	2016	Metal clip
Case 8	79	M	2014	2016	Sponge
Case 9	53	F	2006	2015	Sponge
Case 10	46	F	2014	2015	Whole needle
Case 11	61	F	2013	2014	Sponge
Case 12	56	M	2014	2014	Sponge
Case 13	68	M	2003	2014	Sponge
Case 14	60	F	2013	2014	Sponge
Case 15	89	F	2012	2013	Sponge
Case 16	63	M	2012	2012	Broken central catheter
Case 17	63	M	2011	2012	Broken surgical needle
Case 18	89	M	2011	2012	Broken drill bit
Case 19	57	F	2011	2012	Sponge
Case 20	59	M	2010	2011	Electrostimulator
Case 21	61	M	1983	2011	Sponge
Case 22	55	F	2002	2011	Broken cutter tip
Case 23	61	M	2011	2011	Sponge
Case 24	62	M	2009	2011	Sponge
Case 25	72	M	2011	2011	Sponge
Case 26	88	M	2000	2010	Sponge
Case 27	76	F	1981	2010	Sponge

As mentioned, 27 cases corresponded to medical malpractice claims: 63% of the cases were in the form of C1 (claim directly addressed to the hospital), 21% were C2 (civil mediation), and 17% were C3 (civil procedure).

In the control group, 76% of the cases were directly addressed to the hospital (C1), 16% started as civil mediations (C2), and 8% started as civil procedures (C3).

**Figure 2:**
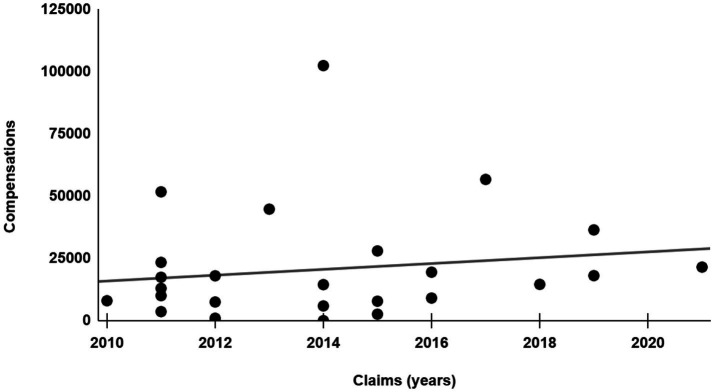
Economic compensation.

Considering only non-fatal events, the mean compensation was €20,695.49 for the RSFB-related cases (Figure 2) and €67,542.26 for the control cases. Among the cases and controls, respectively, 8 and 29% of the total were never compensated.

Regarding the secondary endpoint, considering only non-fatal events, criminal complaints were present in 12% of cases. On the other hand, when limiting our analysis to non-fatal controls with compensations not exceeding €102,481.41, criminal complaints were present in 6% of the total.

## 4 Discussion

RSFB occurrences can pose a serious threat to patient health and safety and raise serious medicolegal concerns. Despite common policies, such as surgical instrument counting, RSFBs are considered “never events,” and thus, it is extremely easy for the claimant to obtain compensation based on the international legal principle “res ipsa loquitur” (18). This principle states that a rebuttable presumption of negligence is allowed if it is proven that the harm would not normally occur without the negligence of the party who has the legal duty to control the most likely cause of the harm (19). In Italy, cases of medical malpractice can be of both criminal and civil interest, even if criminal liability is regulated by a “safe harbor law” (20) that substantially limits it to cases of gross negligence (if the defendant fails to prove compliance with scientific guidelines/best practices). As a result, civil proceedings significantly outnumber criminal cases in the realm of medical malpractice (21).

In our study, we analyzed the claims that occurred at Careggi University Hospital, a large teaching hospital, which is one of the categories of health institutions (teaching hospitals and large hospitals) that have been associated with the highest incidence of RSFBs (22). We found that most cases occurred in middle-aged male patients. These findings are only partially consistent with those of Al-Qurayshi et al., who reported the largest incidence of RSFBs in female patients, with an average age of 50.9 years (23).

In almost 25% of cases, the RSFB is related to orthopedic surgical procedures, maybe because of the significant number of instruments that are generally used in these surgeries, while relatively few cases involve gynecology and abdominal surgeries. In other studies, the most affected disciplines were abdominal surgery, followed by cardiovascular, gynecological, and orthopedic surgeries, with orthopedics prevailing only in the pediatric population (22, 23).

Consistent with previous literature (4), most RSFBs were sponges. However, unlike other reports, most of our cases occurred during elective surgery. The latter difference could be explained by the fact that we selected and included only medicolegal claims related to RSFBs rather than just incident reports. Some cases that occurred during procedures and surgeries performed under emergency conditions may be missing, as patients in these situations might be more inclined to excuse the incident, unlike in planned, routine procedures, where the occurrence of RSFBs is deemed *prima facie*, more deplorable.

The negative social perception of these events is also suggested by the fact that claims related to RSFBs—normalized for economical quantification as performed here—have a double risk of being associated with criminal lawsuits against physicians compared to control cases. Moreover, the RSFB cases were rarely found to correlate with serious health consequences, with only one case in which the incident resulted in the death of the patient. Similarly, Seabra et al. reported that in most of the cases, the only consequence of RSFBs was extended hospitalization, while severe injuries were often temporary, and the death of the patient occurred rarely (0.3%) (24). Therefore, the incidence of litigation due to RSFBs scarcely correlates with the severity of the consequent damage to the patient. As a result, even very minor consequences of RSFBs can give rise to litigation. Despite the low average compensations for the RSFB-related cases compared to the control sample (mean value of 22,327.12 vs. 67,542.26 euros), almost all patients who experienced an RSFB sought compensation. However, they preferred to turn to court settlements or, at least, to mediation provided by third parties in a higher percentage compared to the control sample. This tends to indicate that patients perceive an RSFB as a never event, which requires less justification compared to other wrongful medical care, generates a deeper breach of trust in hospitals and doctors, and renders patients less confident about out-of-court settlements provided directly by hospital committees compared to judicial court trials (17, 25). Furthermore, the incidence of criminal lawsuits was investigated, and it was found to be double in the RSFB cases (12%) compared to the control sample (6%). In Italy, criminal reporting of medical malpractice is mandatory only when improper care could have caused the death of the patient, and the doctor must be investigated for culpable homicide. In cases of culpable personal lesions, when patients experience only temporary or permanent injuries, the report is not mandated by law, and it is the patient’s choice whether to file a criminal lawsuit against the doctor (26). Since we excluded the fatal cases, no mandatory criminal reports or penal proceedings for culpable homicides were included in the study, and the comparison between the RSFB and control samples focused only on the incidence of criminal proceedings voluntarily initiated by the patients. The significantly higher incidence of criminal lawsuits in RSFB cases indicates that the event is likely viewed by the patient as a deep breach of trust in doctors, even if the severity of the actual consequences was mild, as shown by the lower mean compensations compared to the control cases. As a consequence, the cases showed a lower tendency to directly request compensation from the hospital. As previously mentioned, criminal proceedings for RSFBs are particularly complex for the defendant, since these “never events” are often considered evidence of gross negligence. Moreover, hospitals and practitioners risk suffering a significant loss in terms of reputation in RSFB cases, as similar events are heavily publicized and stigmatized (27). Finally, a critical finding was that the incidents were internally reported to the hospital’s designated offices in less than 50% of the cases. This evidence highlights the importance of managing claims as a source of incident reporting but also unveils a reticence about admitting these types of errors, although early reporting and proper intervention are critical factors for patient safety (28). This reticence is likely due to psychological factors, as more than 80% of the involved physicians experience significant distress due to the litigation, which is often seen as indefensible, and the reputational consequences (29).

## 5 Conclusion

To the best of our knowledge, our paper is the first to analyze medical malpractice claims associated with criminal suits related to RSFBs. Our cases, compared to the controls, showed that the health consequences of these “never events” are usually mild, but in almost all the cases (27 out of 28), compensation was requested by the patient. RSFBs are associated with a 2-fold risk of criminal lawsuits and an increased tendency to turn to civil court for compensation instead of the out-of-court scheme offered by the hospital, indicating that these events cause a deeper breach of trust among patients (30). The discrepancy between relatively low health consequences and mean compensation and high litigiousness is likely due to the social stigmatization of this issue. It is recommended to address this through proper risk management strategies and extrajudicial negotiations to contain reputational damage and the psychological distress experienced by the involved professionals.

In the scientific literature, as mentioned, many preventive interventions have been reported and could be introduced to verify their effectiveness. However, our data showed that a pivotal role must be played by risk managers, who must enhance the incident-reporting systems and promote, through internal audits, the disclosure of organizational/individual issues (including communication issues between physicians and patients) and the engagement of operating room personnel. Finally, we believe that investigating this phenomenon internally is of critical economic interest to the institution, particularly in terms of enhancing the reserve fund assessment.

## 6 Limitations

Our study has several limitations. As mentioned, the monocentric study design limited the volume of data, so future multicentric studies are recommended. Moreover, the unpaired sets of the categorical variables with unequal sample sizes prevented us from reliably performing parametric statistical tests to verify whether the variations were statistically significant. At the same time, the sets of continuous variables considered for the *t*-test had different sizes. In general, the main limitation is the small sample size, which stemmed from the fact that, to date, no Italian institution has reported this type of analysis in the scientific literature. Therefore, increasing sample sizes (for instance, by designing multicentric studies) is recommended.

## Data Availability

The original contributions presented in the study are included in the article/supplementary material, further inquiries can be directed to the corresponding author.
